# Age- and gender-based frequency and association of common myeloproliferative mutations in a South African cohort

**DOI:** 10.4102/ajlm.v15i1.2862

**Published:** 2026-01-08

**Authors:** Bathabile Mbele, Kapila Bhowan, Brendon Roets

**Affiliations:** 1 Department of Biomedical Science, Faculty of Health Sciences, University of Johannesburg, Johannesburg, South Africa

**Keywords:** *breakpoint cluster region-Abelson kinase 1*, *calreticulin*, *Janus kinase-2* exon 12, *Janus kinase-2* p.V617F, *myeloproliferative leukaemia virus oncogene*, myeloproliferative neoplasms

## Abstract

**Background:**

Age, gender, and mutation type are key risk factors for myeloproliferative neoplasms (MPNs). Africa remains under-represented in global cancer statistics due to limited population-based genomic data.

**Objective:**

To determine the frequency and demographic associations of common MPN-related genetic abnormalities in the South African population.

**Methods:**

A retrospective cross-sectional analysis of cytogenetic results for *Janus kinase-2* p.V617F (*JAK-2* p.V617F), *Janus kinase-2* exon 12 (*JAK-2* exon 12), *calreticulin* (*CALR*), *myeloproliferative leukaemia virus oncogene* (*MPL*), and *breakpoint cluster region-Abelson kinase 1* (*BCR::ABL1*) was conducted from 01 January 2018 to 31 May 2023. Data were retrieved from the National Health Laboratory Service and analysed for associations with age and gender using Fisher’s Exact Test or Pearson’s Chi-Square Test (*p* < 0.05).

**Results:**

A total of 8934 patient records were analysed; 58% were male patients and 42% female patients, with a mean age of 50 ± 17 years. Among sequence variant changes, 18.2% of MPN cases were positive for *BCR::ABL1*, 8.5% for *JAK-2* p.V617F, 0.5% for *CALR*, 0.04% for *MPL*, and none for *JAK-2* exon 12. *BCR::ABL1* showed equal sex distribution, while *JAK-2* p.V617F increased with age and showed slight female predominance (*p* = 0.002). *CALR* and *MPL* frequencies were too low for meaningful association testing.

**Conclusion:**

*BCR::ABL1* was the most frequent abnormality, especially in younger age groups, whereas *JAK-2* p.V617F was linked to increasing age and female predominance.

**What this study adds:**

MPN genetic testing in South Africa predominantly targeted male patients (ratio 1.4:1). *BCR::ABL1* was the most common abnormality, particularly in individuals aged 18 to 49 years, while *JAK-2* p.V617F showed a slight female predominance (1:1.2).

## Introduction

Myeloproliferative neoplasms (MPNs) are a group of chronic haematological cancers, characterised by the clonal expansion of terminally differentiated myeloid cells.^1^ The World Health Organization and the Polycythaemia Vera (PV) Study Group sub-divided MPNs into either Philadelphia chromosome-positive (Ph+) or Philadelphia chromosome-negative (Ph-) MPNs, based on the presence or absence of the translocation t(9;22)(q34;q11.2).^2^ This specific translocation, referred to as *breakpoint cluster region-Abelson kinase 1* (*BCR::ABL1*), is the only genetic abnormality that causes chronic myeloid leukaemia.^3^ The three most common Ph MPNs include PV, Essential thrombocythaemia (ET) and Primary myelofibrosis (PMF).^4^ Polycythaemia vera is associated with an increased red cell mass and can be attributed to the *Janus kinase-2* p.V617F (*JAK-2* p.V617F) sequence variant change (greater than 95%),^5^ or a *Janus kinase-2* exon 12 (*JAK-2* exon 12) sequence variant change (around 8.3%).^6^ Additionally, the prevalence of *JAK-2* p.V617F is around 55% to 65% among patients with ET and PMF, and are associated with chronic thrombocytosis or fibrosis respectively.^7^ These conditions may be caused by either *JAK-2* p.V617F, *calreticulin* (*CALR*) or *myeloproliferative leukaemia virus oncogene* (*MPL*) sequence variant changes.^8^

Myeloproliferative neoplasms are relatively uncommon, both internationally and within South Africa. An incidence study from 2004 to 2013 reported MPN cases with a frequency of 10% out of 3603 cases from the Eastern Cape province, South Africa.^9^ Myeloproliferative neoplasms are strongly correlated with increasing age, as incidence reports have shown that MPNs are extremely infrequent in the paediatric population, and four to five times greater in geriatric populations.^10^ Age has been linked as a key risk factor for MPN diagnosis and prognosis in certain populations. Māori and Pacific Islanders were diagnosed earlier and passed away at a younger age compared to patients of European descent.^11^ In South Africa, Ph MPNs were most frequently observed within the 55 to 65 years age group, and demonstrated a slight female predominance (1.24:1).^12^ Similarly, Chatambudza et al., in 2021, found that *JAK-2* V617F positive MPNs typically affected older patients (> 60 years of age) with comparable gender ratios.^13^ Previous studies in South Africa obtained the following incidence rates: PV: 97% *JAK-2* p.V617F, 3% triple negative (*JAK-2, CALR, MPL*); chronic myeloid leukaemia: 88% *BCR::ABL1*; ET: 72% *JAK-2* p.V617F, 23% *CALR*, 5% triple negative; PMF: 78% *JAK-2* p.V617F, 16% *CALR*, 6% triple negative.^14,15^

Age and gender have emerged as important risk factors influencing disease progression.^16^ To identify epidemiological patterns and potential at-risk groups, comprehensive databases are required to capture cancer statistics, sequence variant change frequencies, and patient demographics.^17^ Such databases are instrumental in supporting the development of personalised screening, treatment strategies, and in optimising healthcare resource allocation.^17^ Additionally, databases enable comparative analyses across different regions and populations, providing valuable insights into MPN risk factors, aetiology, and disease progression.^18^ Africa remains underrepresented in global cancer statistics, largely because of the limited availability of population-based data, especially from rural settings and the high cost of molecular testing.^19^ Similarly, data on the frequency and demographic distribution of MPN sequence variants within South Africa are scarce and outdated. The aim of this study was to determine the frequency of common MPN-associated genetic abnormalities such as sequence variants (*JAK-2* p.V617F, *JAK-2* exon 12, *CALR*, and *MPL*) and fusions genes (*BCR::ABL1*) for potential relationships with patient demographics (age and gender) within the South African population who are serviced by the National Health Laboratory Service (NHLS).

## Methods

### Ethical considerations

Authorisation to access the NHLS’ testing data were obtained from the data management section of the NHLS, Academic Affairs and Research Unit (reference number: PR2346442; service request number: SR 3764729). Ethical clearance for the study was acquired from the University of Johannesburg’s Faculty of Health Sciences Research Ethics Committee (clearance number: REC-2032-2023). To maintain patient anonymity and adhere to *Protection of Personal Information Act 4 of 2013* standards, the data were de-identified by the NHLS Central Data Warehouse. Access to data were restricted to primary personnel (researchers) and specialist personnel (statistician) who assisted with data analysis. The data were kept secure and confidential in a password-protected folder hosted in the cloud.

### Study design and setting

This study employed a retrospective, cross-sectional design to evaluate both molecular and cytogenetic results of new patients suspected of having an MPN, specifically *JAK-2* p.V617F, *JAK-2* exon 12, *CALR, MPL* and *BCR::ABL1*, as well as patient demographics variables (age and gender). All data were obtained from patients tested by the NHLS, South Africa, between 01 January 2018 and 31 May 2023. This study design and use of secondary data facilitated a rapid, cost-effective and comprehensive assessment of the frequency and demographic associations of common MPN sequence variants within the South African population.

### Study population and sampling strategy

The data were extracted from the Central Database Warehouse of the NHLS, a national pathology network that provides diagnostic services to more than 80% of the South African population.^20^ The data set included all samples tested by the NHLS for the specified genetic targets between 01 January 2018 and 31 May 2023 across multiple NHLS molecular laboratories (Charlotte Maxeke, Dr George Mukhari, Groote Schuur, Inkosi Albert Luthuli, Tshwane, Tygerberg and Universitas Academic Hospital laboratories) in South Africa. The search criteria and subsequent extraction utilised the following key words: ‘MPNs, PV, ET, PMF, *JAK-2* p.V617F, *JAK-2* exon 12, *CALR, MPL*, and *BCR::ABL1*’. To avoid duplication, the request specified that only a patient’s initial visit should be extracted. Only patients aged 18 years or older, with complete demographic records (age and gender), were included. Patients younger than 18 years, those with incomplete records, and those diagnosed with rare MPN genetic abnormalities (other than *BCR::ABL1, JAK-2* p.V617F, *JAK-2* exon 12, *CALR*, and *MPL*) were excluded.

### Bias

The NHLS operates under the South African National Accreditation System accreditation, requiring strict adherence to internal and external quality control programmes. This ensures accuracy, reliability and validity of molecular test results. Sampling bias was minimised by including all eligible cases that met the inclusion criteria during the defined study period. Because this was a retrospective study, the sample size could not be predetermined; however, the final data set was sufficiently large to reduce statistical bias and enhance representativeness of current MPN variant distribution in South Africa.

### Data analysis

De-identified data were obtained in Microsoft Excel (Microsoft, Redmond, Washington, United States) format from the NHLS Central Data Warehouse. The records were manually verified and sorted to exclude incomplete entries. The data were categorised by year, gender, age and mutational status to facilitate statistical analysis. The data set was imported into the IBM^®^ SPSS^®^ Statistics program, version 29 for analysis (IBM Corporation, Armonk, New York, United States). Descriptive statistics were presented as frequencies and percentages in tables and graphs. Associations between categorical variables were assessed using Fisher’s Exact Test for 2 x 2 tables and the effect sizes were determined using Phi (small < 0.3, medium 0.3–0.5, large > 0.5). Alternatively, the Pearson’s Chi-Square Test was used for larger tables and the effect sizes were reported using Cramer’s *V*. Statistical significance was set at *p* < 0.05. All analyses were independently verified by a qualified, unbiased statistician.

## Results

### Participants

A total of 11 384 patient records was extracted and reviewed for the period 01 January 2018 to 31 May 2023. However, only 8934 patient records met the inclusion criteria and were retained for statistical analysis, while 2450 patient records were excluded because of incomplete demographic information or patient age below 18 years.

### Descriptive data

**FIGURE 1:**
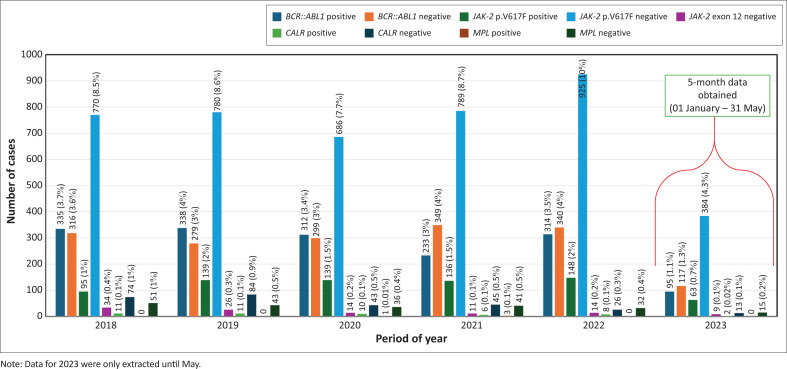
Haematology-based molecular and genetic results by year across South Africa from 01 January 2018 to 31 May 2023. The Pearson’s Chi-Square test revealed a statistically significant difference in *BCR::ABL1* over the years (*p* < 0.001), with a small effect size of Cramer’s *V* = 0.097. In contrast, no statistically significant differences were observed for *JAK-2* p.V617F (*p* = 0.565), *CALR* (*p* = 0.519), and *MPL* (*p* = 0.108), with all three showing no effect size. Additionally, no positive cases were identified for *JAK-2* exon 12.

An average of 1489 samples were tested annually between 01 January 2018 and 31 May 2023 across the seven NHLS laboratories included in this study (Figure 1). The three laboratories with the highest testing volumes were Charlotte Maxeke, Groote Schuur, and Inkosi Albert Luthuli (Table 1). Male patients comprised 5175 (58%) of the total cohort, while 3759 (42%) were female patients, yielding a male-dominated testing ratio of 1.4:1. The mean patient age at testing was 50 ± 17 years.

**TABLE 1:** Geographical overview of the results from different laboratories across South Africa from 01 January 2018 to 31 May 2023.

Laboratory name	Laboratory location	*BCR::ABL1*		*JAK-2* p.V617F		*JAK-2* exon 12		*CALR*		*MPL*		Total
		Pos	Neg	Pos	Neg	Pos	Neg	Pos	Neg	Pos	Neg	
Charlotte Maxeke Hospital	Gauteng	946	806	230	1891	0	0	15	58	0	4	**3950**
Dr George Mukhari Hospital	Gauteng	94	112	0	0	0	0	0	0	0	0	**206**
Groote Schuur Hospital	Western Cape	269	401	218	1056	0	0	12	23	0	51	**2030**
Inkosi Albert Luthuli Hospital	KwaZulu-Natal	206	218	133	469	0	15	8	49	2	43	**1143**
Tshwana Academic Hospital	Gauteng	0	0	51	283	0	0	0	0	0	0	**334**
Tygerberg Hospital	Western Cape	0	0	80	369	0	80	10	29	0	36	**604**
Universitas Academic Hospital	Free State	112	165	44	266	0	13	3	46	2	16	**667**

**Total**	**-**	**1627**	**1702**	**756**	**4334**	**0**	**108**	**48**	**205**	**4**	**150**	**8934**

Note: Charlotte Maxeke Hospital laboratory also processed samples from Limpopo and Chris Hani Baragwanth Academic Hospital. The Dr George Mukhari Hospital laboratory serviced Limpopo, Mpumalanga, and the North West provinces. The Groote Schuur Hospital laboratory assisted the Northern Cape and Western Cape provinces. During the study period Tygerberg Hospital’s *BCR::ABL1* testing was conducted at the Groote Schuur Hospital laboratory. The Tygerberg Hospital laboratory also included samples from the Northern Cape and Western Cape provinces. The Universitas Academic Hospital laboratory also processed samples from the North West province.

Pos, positive; Neg, negative; *BCR::ABL1, breakpoint cluster region-Abelson kinase 1; JAK-2* exon 12, *Janus kinase-2* exon; *JAK-2* p.V617F, *Janus kinase-2* p.V617F; *CALR, calreticulin; MPL, myeloproliferative leukaemia virus oncogene*.

### Outcome data

**FIGURE 2:**
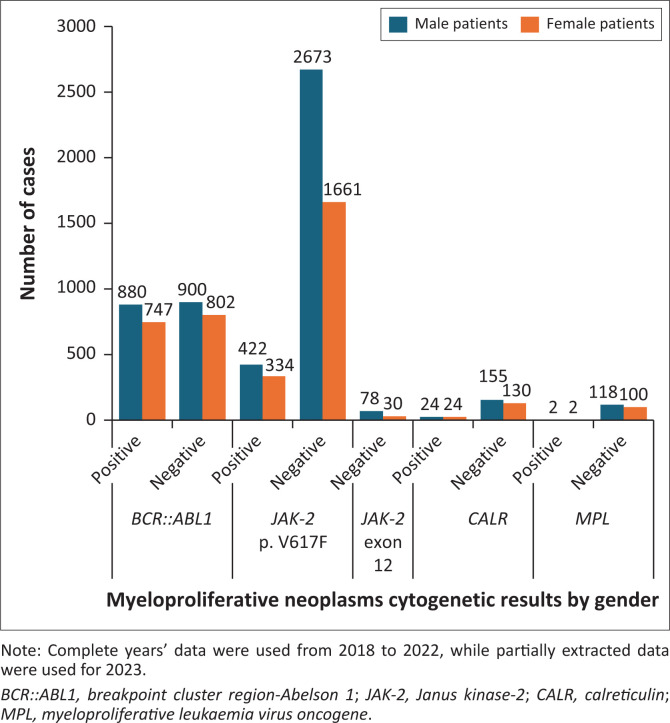
Haematology based molecular and genetic results by gender across South Africa from 01 January 2018 to 31 May 2023.

**FIGURE 3:**
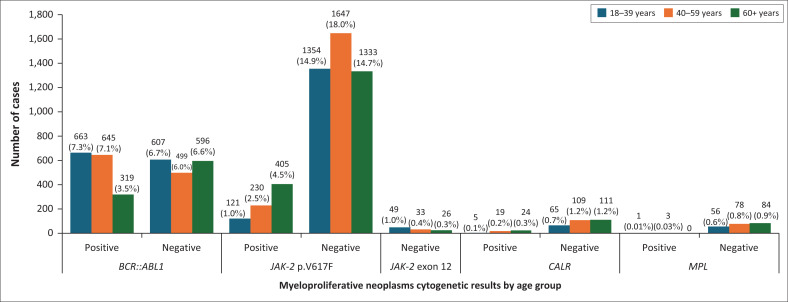
Haematology-based molecular and genetic results by age group across South Africa from 01 January 2018 to 31 May 2023.

The sequence variant changes tested for were tallied as follows: 18.2% of the cases (*n* = 1627) were positive for *BCR::ABL1*, 8.5% were positive for *JAK-2* p.V617F (*n* = 756), 0.5% were positive for *CALR* (*n* = 48), 0.04% were positive for *MPL* (*n* = 4), and no sequence variant changes were found in any of the cases for *JAK-2* exon 12 (*n* = 0). The *BCR::ABL1* fusion gene showed an equal gender incidence; *JAK-2* p.V617F showed a slight female predominance, and *CALR* and *MPL* association testing was limited by a small number of positive cases. The *BCR::ABL1* fusion gene was frequently diagnosed in the younger age groups of 18 to 39 years (52.2%) and 40 to 59 years (56.4%). The *JAK-2* p.V617F sequence variant change positivity increased with age; the 60+ years age group (23.3%) had the highest incidence. Similarly, the *CALR* sequence variant change positivity increased with age, the 60+ years group had the highest incidence (17.8%). *Myeloproliferative leukaemia virus oncogene* association testing was limited by a small sample size. The associations between year, age, gender and sequence variant changes status are summarised in Table 2 and illustrated in Figure 1, Figure 2, and Figure 3.

**TABLE 2:** Associations between year, age, gender and sequence variant changes status across South Africa from 01 January 2018 to 31 May 2023.

Comparison	Significance	Effect size
Year versus *BCR::ABL1*	Pearson’s Chi Square test ≤ 0.001***	Cramer’s *V* = 0.097†
Year versus *JAK-2* p.V617F	Pearson’s Chi Square test = 0.565	No effect size
Year versus *CALR*	Pearson’s Chi Square test = 0.519	No effect size
Year versus *MPL*	Pearson’s Chi Square test 0.108 Cannot use the *p*-value, it can be biased. Larger than 20%, cannot perform the test (50%).^21^	No effect size
Gender versus *BCR::ABL1*	Fisher’s Exact Test = 0.487	No effect size
Gender versus *JAK-2* p.V617F	Fisher’s Exact Test = 0.002**	Phi = 0.043†
Gender versus *CALR*	Fisher’s Exact Test = 0.640	No effect size
Gender versus *MPL*	Fisher’s Exact Test = 1.000	No effect size
Age versus *BCR::ABL1*	Pearson’s Chi Square test ≤ 0.001***	Cramer’ *V* = 0.176†
Age versus *JAK-2* p.V617F	Pearson’s Chi Square test ≤ 0.001***	Cramer’ *V* = 0.177†
Age versus *CALR*	Pearson’s Chi Square test = 0.119	No effect size
Age versus *MPL*	Pearson’s Chi Square test = 0.202 Cannot use the *p*-value, it can be biased. Larger than 20%, cannot perform the test (50%).^21^	No effect size

Note: *JAK-2* exon 12 had no positive cases. Please see the full reference list of this article for details on the article cited: Mbele B, Bhowan K, Roets B. Age- and gender-based frequency and association of common myeloproliferative mutations in a South African cohort. Afr J Lab Med. 2025;14(1), a2862. https://doi.org/10.4102/ajlm.v14i1.2862.

** , significant difference at *p* < 0.01;

*** , significant difference at *p* < 0.001.

† , small effect.

A statistical analysis employing Fisher’s Exact test indicated a significant difference in *JAK-2* p.V617F based on gender (*p* = 0.002), with an effect size of Phi = 0.043. Conversely, no significant differences were found for *BCR::ABL1* (*p* = 0.487), *CALR* (*p* = 0.640), and *MPL* (*p* = 1.000), all of which demonstrated no effect size. Furthermore, there were no positive cases detected for *JAK-2* exon 12.

The analysis performed using the Pearson’s Chi Square test indicated a statistically significant difference in *BCR::ABL1* with respect to age (*p* < 0.001), exhibiting a small effect size (Cramer’s *V* = 0.176). Similarly, a significant difference was found for *JAK-2* p.V617F in relation to age (*p* < 0.001), with a small effect size (Cramer’s *V* = 0.177). Conversely, no statistically significant differences were detected for *CALR* (*p* = 0.119) and *MPL* (*p* = 0.202), both of which demonstrated no effect size. Furthermore, no positive cases were recorded for *JAK-2* exon 12.

## Discussion

Myeloproliferative neoplasm diagnosis, treatment and management have benefitted from the identification of common driver gene mutations. The incorporation of driver gene mutations as a primary criterion by the World Health Organization highlights the critical need to conduct testing for these genetic markers to facilitate the diagnosis, classification, and prognostication of various MPN subtypes.

The NHLS distributes nationally collected samples to designated testing laboratories across all nine provinces for specific assays and services to optimise cost and workload management.^20^ Genetic (molecular/cytogenetics) testing facilities are strategically located in Gauteng (Charlotte Maxeke Hospital, Dr George Mukhari Hospital and Tshwane Academic Hospital), Western Cape (Groote Schuur Hospital and Tygerberg Hospital), Free State (Universitas Academic Hospital), and KwaZulu-Natal (Inkosi Albert Luthuli Hospital).^22^ Consequently, the geographical distribution of results reflects the testing site allocation determined by NHLS internal policies and does not correspond to the patient’s residential location, limiting interpretation of genetic abnormality distribution nationally. Gauteng (Charlotte Maxeke Hospital and Chris Hani Baragwanath Hospital laboratories) processed approximately double the testing volume (2121 samples) for *JAK-2* p. V617F compared to the Western Cape (Groote Schuur Hospital) (1274 samples). Despite this difference, both regions reported similar numbers of positive cases; this regional variation warrants further investigation. One possible contributing factor may be demographic variation, as the Western Cape has a higher female population, especially in the older age groups, compared to Gauteng.^23,24^ A previous study has also reported a slight female predominance for the *JAK-2* p. V617F sequence variant.^12^

The *BCR::ABL1* and *JAK-2* p.V617F abnormalities were tested more frequently (93%), as these serve as first-line tests for patients suspected of having MPNs.^25^ The *BCR::ABL1* fusion gene was the most identified aberration (18.2%). The *JAK-2* p.V617F sequence variant change is the most frequently identified in Ph MPNs globally.^13^ Similarly, *JAK-2* p.V617F was the most commonly identified Ph MPN sequence variant (8.5%) in this study and second in frequency to the *BCR::ABL1* fusion gene. Shires et al. in 2023, reported a 23% frequency for the *JAK-2* p.V617F sequence variant change, based on a retrospective study from 2011 to 2018 at the Groote Schuur Hospital Molecular laboratory in the Western Cape.^15^ The study of Shires et al. in 2023 only focused on Ph MPNs from a single testing facility and a smaller sample size, because of the higher frequency.

In 2022, Gul et al. reported that an incidence of 96% of cases showed *JAK-2* sequence variant change positivity.^26^ A study conducted in Eastern Anatolia by Gulbay et al. in 2019 found that 22.6% of the patients had the *JAK2* p.V617F sequence variant, with 33.3% of women and 19.2% of men testing positive.^27^ Both studies showed a high percentage of *JAK-2* p.V617F sequence variant changes, using a smaller sample size compared to this study. The Gul et al. study in 2022 exclusively examined the Ph- patients, skewing their results towards a higher percentage of *JAK-2* p.V617F.

The *JAK-2* exon 12, *CALR* and *MPL* sequence variant changes are only tested when *BCR::ABL1* and *JAK-2* p.V617F test results are negative. Testing is prioritised in this way to reduce costs and efficiently manage limited healthcare resources.^28^ In this study, no patients with *JAK-2* exon 12 sequence variant change were found. This can be attributed to the rarity of *JAK-2* exon 12 sequence variant change and their occurrence in younger patients, with PV or ET being linked to most reported cases.^29^ The *MPL* sequence variant change was also rare, with only four (0.04%) cases being positively identified in this study. Similarly, Shires et al.’s study in 2023 found three (6%) *MPL* sequence variant changes, and noted that *MPL* are generally considered to be rather uncommon events, with frequencies between 1% and 8%.^15^ This is supported by studies of Chinese and Korean populations that found 1% to 4% of *MPL*-positive individuals, as well as Iranian and Turkish populations with 4% to 6%.^30^ Ethnic differences might be the cause of this slight discrepancy.^31^ In the four *MPL*-positive patients identified in our study, only the *MPL*-W515L sequence variant changes were detected; no *MPL*-W515K sequence variant changes were identified. It was discovered that *MPL*-W515A/R sequence variant changes were considerably less common than *MPL*-W515L/K sequence variant changes.^32^

The so-called ‘triple-negative’ MPNs refer to MPN patients without evidence of any of the three major sequence variant changes (*JAK-2, CALR* and *MPL*).^33^ Identifying triple-negative patients is crucial, since triple-negative patients tend to have a worse prognosis.^34^ The extracted data contained a single confirmed MPN case within the study cohort that was classified as triple-negative. This rare condition often requires further molecular testing to identify other genetic changes, such as those in the SH2B3 (LNK) gene.^35^ Testing reflexes for other variants is not usually performed, except when instructed by the pathologist or clinician.^35^ This can affect the final result and represents another limitation of the study. In 2018, De Roeck et al. detected coexisting driver sequence variant changes (*JAK-2* p.V617F + *BCR::ABL1: n* = 8; *CALR* + *BCR::ABL1: n* = 1; *JAK-2* p.V617F + *MPL: n* = 1; *JAK-2* p.V617F + *CALR: n* = 1),^36^ whereas no double sequence variant changes were identified in our study, highlighting the limitation of using retrospective data compared to prospective studies in which patients can be traced and further testing can be performed. Additionally, some sequence variant changes may impact the same gene, such as *MPL*, but their position may differ, making it difficult to detect and report. It is still unclear whether the clinical characteristics and prognosis of patients with dual sequence variant changes vary from those of patients with a single sequence variant change or no sequence variant changes, despite the fact that the study by Lundberg et al. suggested that patients with two or more sequence variant changes typically have a poor prognosis.^37^

When using large data sets (sample sizes), small differences between groups might become statistically significant. Therefore, it is important to determine the effect size of the significance.^35^ The *BCR::ABL1* fusion showed a significance (*p* < 0.001) when compared to year. However, the effect size of the significance was small (odds ratio = 0.097). Therefore, no significant correlation between year and *BCR::ABL1* results could be identified. However, both *BCR::ABL1* testing frequency, and the number of positive cases, declined between 2020 and 2021. This could be attributable to the peak of the coronavirus disease 2019 pandemic in South Africa, highlighting a shift in pathology testing and patient accessibility to healthcare during ‘lockdown’ periods.^38^ Similarly, there was no significant association between year and the other MPN sequence variant changes.

No significant difference could be detected between *BCR::ABL1* and gender, indicating an equal incidence between male and female patients (1:1). Similarly, the research conducted by Azad et al. in 2018 revealed no correlation with gender,^39^ however the findings of the Ghalesardi et al. study in 2021 revealed a slightly male/female ratio that varied from 1.3 to 1.8.^40^ The *JAK-2* p.V617F sequence variant change showed a small association (*p* = 0.002; odds ratio = 0.043) with gender, indicating a slight female predominance (1:1.2).

Because women are affected more often than men, there is evidence that hormones and reproductive history may have a role in the risk of haematologic malignancies, particularly non-Hodgkin lymphoma.^21^ However, there is a shortage of information about the risk of myeloid malignancies, and no studies that directly address the risk of MPNs.^41^ In comparison, two studies conducted in South Africa, by Mayet in 2015,^12^ and Chatambudza in 2021,^13^ revealed that female patients are slightly more dominant than male patients, with a ratio of 1.24:1,^12^ while the other study demonstrated an equal gender ratio.^13^ The *CALR* sequence variant changes showed no significant association with gender, but there was a slight female predominance in the number of positive cases. However, this comparison was limited by the low frequency of positive cases and small sample size, compared to the larger sample sizes found in *BCR::ABL1* and *JAK-2* p.V617F. Similarly, *MPL* sequence variant changes could not be associated with gender, as there were of only four positive cases.

Three age-groups (18 to 39 years, 40 to 59 years, and 60+ years) were created to ensure that the group sizes were similar and acceptable for statistical analysis. Globally, *BCR::ABL1* (chronic myeloid leukaemia) was associated with older age groups (> 60).^42^ The *BCR::ABL1* fusion showed a significant association with age, but the effect size was small (*p* < 0.001; odds ratio = 0.176). More positive cases were identified in the younger age groups (18 to 39 years [52.2%] and 40 to 49 years [56.4%]), compared to the older age group of 60+ years (34.9%). Similarly, several studies conducted in South Africa and Africa placed the mean age of diagnosis between 38 and 49 years.^14,38,43,44,45^ However, a study performed at Universitas Academic Hospital by Sikhipha et al. in 2020, discovered that patients as young as 16 years had been diagnosed with chronic myeloid leukaemia,^46^ suggesting that the mean age varies. This might be attributable to a younger age structure seen in South African demographics, as opposed to older populations seen in developed countries, or unknown environmental exposures.^43^ Furthermore, it is possible that the elderly patients (who are over 60) had an earlier diagnosis, indicating efficient screening in South Africa.^7^ Since our study focused on individuals aged 18 years and older, it is important to note that established diagnostic and treatment guidelines for MPNs have historically been tailored for adults rather than children.^47^ Sikhipha et al.’s 2020 study indicates that MPNs can occur in younger populations, but for these age groups, the understanding is still evolving.

In contrast to *BCR::ABL1, JAK-2* p.V617F sequence variant changes showed a significant association with age (*p* < 0.001; odds ratio 0.177), with a trend of increasing frequency with increasing age. The highest incidence was in the 60+ years age group (23.3%). Similarly, other SA-based studies have identified the mean age of diagnoses to be 52.8 and 59.7.^15^ However, these studies reported their results without creating age categories. No significant association was seen between *CALR* and age because of the small sample size. The data demonstrated a trend of increasing with age, as the 60+ years group showed the highest incidence (17.8%). Najim et al.’s investigation demonstrated that, in contrast to this earlier discovery, the majority of *CALR* positive MPNs are found in younger individuals.^48^ No significant association could be made between *MPL* and age because of the small sample size. However, the majority of the positive cases presented in the 40 to 59 years age group.

### Limitations of the study

The use of de-identified data rendered patient tracing impossible; therefore, it was not possible to identify the coexistence of sequence variant changes, and the identification of triple-negative cases was impaired. Data for 2023 were only extracted for the first half of the year, thus limiting the sequence variant change frequency and statistics for 2023. This study was limited to only age and gender, but race is also an important prognostic factor to consider. We acknowledge that the different NHLS laboratories utilise varied testing platforms and methodologies (Online Supplementary Document 1), which may result in differences in assay sensitivity and detection ranges. These methodological variations could potentially influence the observed mutation frequency rates reported in this study. The study was limited to data from the public sector but is representative of the incidence in SA considering that the NHLS offers its service to 80% of SA’s population.^49^ Lastly, this study did not review the clinical information or morphological results of the patients, therefore it was not possible to determine whether any acute lymphoid leukaemia patients with a t(9;22) were included in the data set.

### Conclusion

In conclusion the study findings were consistent with global and regional trends. The frequent testing and identification of *BCR::ABL1* fusions and *JAK-2* p.V617F sequence variant changes emphasise their importance as first-line diagnostic markers. The *BCR::ABL1* fusion was the most commonly identified genetic abnormality and was more prevalent in younger age groups. The *JAK-2* p.V617F sequence variant was the predominant mutation in Ph-negative MPNs and showed increasing frequency with age and a slight female predominance. However, MPN testing frequency was skewed towards male patients; therefore, it is recommended that more women be screened for *JAK-2* p.V617F, especially in older populations if clinically indicated. The rarity of *JAK-2* exon 12, *CALR* and *MPL* sequence variant changes align with international data, though small sample sizes limited detailed statistical analysis. This study highlighted the challenges in identifying ‘triple-negative’ cases, especially when using retrospective data. No significant gender associations were found for most sequence variant changes. Age trends suggested that demographic and environmental factors, coupled with South Africa’s unique population structure, may influence sequence variant change distribution. Further research is required, since cancer statistics in Africa and South Africa are limited by a lack of reliable population-based data. Further research should focus on ethnicity and environmental factors influencing MPN epidemiology in South Africa. Addressing the limitations of retrospective analysis and patient tracking will be crucial for advancing our understanding of MPNs and optimising patient outcomes.
